# The Role of the Two-Component System BaeSR in Disposing Chemicals through Regulating Transporter Systems in *Acinetobacter baumannii*

**DOI:** 10.1371/journal.pone.0132843

**Published:** 2015-07-10

**Authors:** Ming-Feng Lin, Yun-You Lin, Chung-Yu Lan

**Affiliations:** 1 Department of Medicine, National Taiwan University Hospital Chu-Tung Branch, Hsin-Chu City, Taiwan; 2 Institute of Molecular and Cellular Biology, National Tsing Hua University, Hsin-Chu City, Taiwan; 3 Department of Life Science, National Tsing Hua University, Hsin-Chu City, Taiwan; Tianjin University, CHINA

## Abstract

Bacterial two-component regulatory systems (TCSs) facilitate changes in gene expression in response to environmental stimuli. TCS BaeR regulons influence tigecycline susceptibility in *Acinetobacter baumannii* through positively regulating the pump genes *adeA* and *adeB*. In this study, we demonstrate that an additional two transport systems, AdeIJK and MacAB-TolC, are also regulated by BaeSR. In the wild type and clinical tigecycline-resistant *A*. *baumannii* strains, gene expression of AdeIJK and MacAB-TolC increased after tigecycline induction, implicating their importance to tigecycline resistance in addition to AdeABC. Phenotypic microarray results showed that *A*. *baumannii* is vulnerable to certain chemicals, especially tannic acid, after deleting *baeR*, which was confirmed using the spot assay. The wild-type strain of *A*. *baumannii* also exhibited 1.6-fold and 4.4-fold increase in gene expression of *adeJ* and *macB* in the medium with 100 μg/mL tannic acid, but the increase was fully inhibited by *baeR* deletion. An electrophoretic motility shift assay based on an interaction between His-BaeR and the *adeA*, *adeI* and *macA* promoter regions did not demonstrate direct binding. In conclusion, *A*. *baumannii* can use the TCS BaeSR in disposing chemicals, such as tannic acid and tigecycline, through regulating the efflux pumps.

## Introduction

Efflux pumps actively export antibiotics from the bacterial cell and are, thus, one of the mechanism that contribute to multidrug resistance in bacteria [1]. Four categories of efflux pumps, including the resistance-nodulation-cell division (RND) superfamily, the major facilitator superfamily (MFS), the multidrug and toxic compound extrusion (MATE) family and the small multidrug resistance (SMR) family of transporters, reportedly relate to antimicrobial resistance in *A*. *baumannii* [2,3]. CraA [4] and Tet [2], belonging to the MFS efflux pumps, are conferring to chloramphenicol and tetracycline resistance respectively. AbeM [5], the only efflux pump of the MATE family described so far in *A*. *baumannii*, is shown to extrude aminoglycosides, fluoroquinolones, chloramphenicol, and trimethoprim. AbeS [6], a novel SMR transporter, confers low level resistance to several antimicrobial agents. Of these four different pump categories, the RND superfamily plays the most important role in multidrug resistance. AdeABC is the first well-characterized RND-type efflux pump in *A*. *baumannii* and is associated with lower cell susceptibility to several antimicrobials, including tigecycline [7,8]. Inactivation of an additional two RND-type pumps, AdeFGH [9] and AdeIJK [10–12], also indicates that they contribute to multidrug resistance in *A*. *baumannii*. However, efflux pumps not only confer resistance to certain classes of antibiotics but can also export other natural substances or chemical compounds, indicating that they might play a role in allowing bacteria to survive in their ecological niche [13]. Although the ability of efflux pumps, such as AmvA [14], AbeM [5], and AbeS [6], to dispose of certain chemicals in addition to antibiotics has been described in previous studies of *A*. *baumannii*, the complete picture of this phenotype and its regulatory mechanisms are still unclear.

Previous studies have identified local or global regulators that are involved in efflux gene expression [3]. The best studied example of local regulators in *A*. *baumannii* is the AdeRS TCS, which is a positive activator for the AdeABC efflux pump [15]. Point mutations in AdeS and AdeR or AdeS truncation due to ISAba1 insertion may be related to AdeABC overexpression, which leads to multidrug resistance [15,16]. Another example of a local regulator for an RND-type pump in *A*. *baumannii* is AdeN, which is a TetR-type regulator responsible for regulating AdeIJK expression [17]. The expression of various efflux pumps is also controlled by different global regulators. In *Escherichia coli*, general stress-induced *acrAB* pump gene transcription is primarily mediated by a global regulator pathway [18]. In our previous study, we proposed BaeSR, which is an envelope system that responds to stress from external stimuli, as a global regulator to influence *adeAB* transcription and, thus, tigecycline susceptibility in *A*. *baumannii* [19].

Environmental stress, which damages the outer membrane or disrupts perisplasmic homeostasis in Gram negative bacteria, can stimulate the envelope stress response (ESR) [20]. Five extracytoplasmic stress response pathways have been described for *E*. *coli*, including BaeSR, which belongs to a TCS [21]. The main function of the Bae response is to upregulate efflux pump expression in response to specific envelope-damaging agents [22]. Indole, flavonoids, and sodium tungstate are novel BaeSR response inducers [22,23]. The Bae regulon is involved in defenses to zinc toxicity [24], novobiocin and deoxycholate resistance [25], and condensed tannis resistance [26]. A phenotype microarray analysis using *E*. *coli* TCS gene mutants showed increased sensitivity in the *baeSR* mutant to myricetin, gallic acid, nickel chloride and, especially, sodium tungstate [27]. In *Salmonella typhimurium*, the Bae TCS increases multidrug and metal resistance by inducing the AcrD and MdtABC drug efflux systems [28]. A genome-wide analysis of *E*. *coli* gene expression showed that BaeR overproduction activates genes involved in multidrug transport, flagellum biosynthesis, chemotaxis, and maltose transport [29]. In *A*. *baumannii*, an LPS-deficient strain showed increased expression of genes that encode BaeS/R orthologs and of genes that encode MDR-associated proteins, such as *macAB-tolC and adeIJK* [30].

However, the relationship between BaeSR and certain transporter genes, such as *adeIJK* and *macAB-tolC* is not fully understood in *A*. *baumannii*. Because *A*. *baumannii* is exquisite in adapting its environment and coping with external stress, especially in the hospitals, this study is aimed at understanding the role of BaeSR as a stress response system for disposing chemicals through regulating transporter genes in *A*. *baumannii*.

## Materials and Methods

### Bacterial strains, growth conditions, antimicrobial susceptibility test and DNA manipulation

The bacterial strains used in this study are listed in Table 1. The cells were commonly grown at 37°C in LB broth and agar. For inducing tigecycline resistance, serial passaging was performed as our previous study [19]. To determine the minimal inhibitory concentration (MIC) of tigecycline, a broth microdilution method according to the 2012 CLSI guidelines [31] was used. The MIC was defined as lowest tigecycline concentration that completely inhibited bacterial growth, and bacterial growth was determined by unaided eyes and by measuring optical density (OD) using a spectrophotometer. The provisional MIC breakpoints for tigecycline are ≤2, 4, and ≥8 μg/mL to designate susceptible, intermediate, and resistant strains, respectively [32]. *A*. *baumannii* genomic DNA was extracted as described previously [33]. The DNA was PCR-amplified with a Hybaid PXE 0.2 HBPX02 Thermal Cycler (Thermo Scientific, Redwood, CA) using Pro*Taq* DNA Polymerase (Protech, Taipei, Taiwan) or the KAPA HiFi PCR Kit (Kapa Biosystems, Boston, MA). The DNA fragments were extracted from agarose gels and purified as previously described [19]. The PCR products were verified by DNA sequencing.

**Table 1:** Bacterial strains, plasmids and primers used in this study.

Strains		Relevant feature(s)	Source or reference
*A*. *baumannii* strains	ATCC 17978	Wild-type strain	ATCC
	AB1026 (Δ*baeR*::*kan* ^*r*^)	Derived from ATCC 17978. *baeR* mutant obtained by *kan* ^*r*^ gene replacement	[19]
	AB1027	AB1026 *baeR*::pWH1266	[19]
	AB1028	ATCC 17978 *baeR*::pWH1266	[19]
	ABtc	Induced tigecycline resistant ATCC 17978	[19]
	ABtcm (Δ*baeR*::*kan* ^*r*^)	Derived from ABtc. *baeR* mutant obtained by *kan* ^*r*^ gene replacement	[19]
	ABhl1	Tigecycline resistant clinical isolate	[19]
	ABhl1tc	Clinical isolate with induced high tigecycline resistance	This study
*E*. *coli* strains	BL21 (DE3) pLysS	As a competent cell that allow high-efficiency of protein expression	Novagen
	BL21 His-BaeR	BL21 (DE3) pLysS carrying plasmid pET-23a-His-BaeR	This study
**Plasmids**		**Relevant feature(s)**	**Source or reference**
pET-23a(+)		Subcloning vector with a T7 promoter, N-terminal T7 tag and C-terminal 6xHis tag; *amp* ^*r*^	Novagen
pET-23a(+)-His-BaeR		pET-23a(+) carrying *A*. *baumannii* ATCC 17978 A1S_2883	This study
**Primers**		**Relevant feature(s)**	**Source or reference**
pm_*adeA*_F		GCCTTCACGTTTTAAATA	This study
pm_*adeA*_R		CCTAGTGAGTTTTTGATG	This study
pm_*adeI*_F		ATTTTATCTAAACGAGGTGG	This study
pm_*adeI*_R		TTTCTGAGCAGCAGCAGC	This study
pm_*macA*_F		GGCAGCCAAATCATTTGC	This study
pm_*macA*_R		CTTTTTAACCTGACCAGATACCTG	This study
pET_check_F		CTCGATCCCGCGAAATTA	This study
pET_check_R		GCAGCCAACTCAGCTTCC	This study
pET_baeR_Nhe1_ F		AAAGCTAGCCACCACCACCACCACCACGGTATGTTTCATGATGG	This study
pET_baeR_Xho1_R		TAAACCCTCGAGTTATTCTTCTGGATATTCGAAGCGATAACCTACTCC	This study
q*baeR*_F		TGACAGCACGTACCGAAGAAA	[19]
q*baeR*_R		CATAATCATCTGCCCCCATGT	[19]
q*adeB*_F		ACAAGACCGCGCTAACTTAGGT	[19]
q*adeB*_R		TGCCATTGCCATAAGTTCATCT	[19]
q*adeI*_F		GGCCAATCTGGTCGTTCTTC	This study
q*adeI*_R		CGGGTCAGTCTGGTTTGCA	This study
q*adeJ*_F		AGCTGGTGCTATGGGCGTTA	This study
q*adeJ*_R		GCCACCCCATGCAATACG	This study
q*adeK*_F		TTCCAACAATCGGAGCAAGTG	This study
q*adeK*_R		TTATTCGGATCACGGCTTTGA	This study
q*tolC*_F		CTTACGGACCAGCTCTAGTGTTTCT	This study
q*tolC_*_R		CGCACTGTCATCTCCCGAAT	This study
q*macB*_F		AATGAATGGCGGCGATGTA	This study
q*macB*_R		GTGAATCGAGTGCCCCTGTT	This study
q*macA*_F		TTGGTTCCATCTTCTGCGTTAA	This study
q*macA*_R		GCCGATTGCCCCTGTTT	This study
q16s rRNA_F		AGCATTTCGGATGGGAACTTTA	[19]
q16s rRNA_R		GTCGTCCCCGCCTTCCT	[19]

### RNA isolation and quantitative reverse transcription (qRT)-PCR

Total RNA was isolated by the phenol-chloroform-isoamyl alcohol (PCIA) method. Briefly, the *A*. *baumannii* ATCC 17978 strain was grown overnight in LB broth at 37°C, 220 rpm for 16 hours. The overnight cultures (OD_600_ ~6.5) were sub-cultured at a 1:100 dilution in 25 mL fresh LB medium in the absence of tigecycline. The cells were grown to mid-log phase (the OD_600_ values for ATCC 17978, AB1026, AB1027, AB1028, ABhl1 and ABhl1tc were all ~3 and the OD_600_ values for both ABtc and ABtcm were ~2) and harvested by centrifugation at 4°C. The cell pellets were resuspended in 200 μL ice-cold lysis buffer (0.1 M Tris-Cl [pH 7.5], 0.1 M LiCl, 0.01 M ethylenediaminetetraacetic acid [pH 8.0], 5% sodium dodecyl sulfate [SDS], and 2% β-mercaptoethanol). Then, 200 μL ice-cold PCIA ([25:24:1], pH 4.5) was added. The cells were lysed by vortexing for 2 minutes. Supernatants were collected by centrifugation, extracted with 200 μL ice-cold PCIA. This step was repeated three times. Total RNA was precipitated with ethanol at −80°C overnight and collected by centrifugation at maximum speed for 5 minutes and dissolved in dissolved in 25–100 μL RNase-free water. DNA contaminants were removed using Ambion TURBO DNase (Life Technologies, Grand Island, NY). For cDNA synthesis, RNAs were reverse transcribed using High-Capacity cDNA Reverse Transcriptase Kit (Applied Biosystems). The cDNA samples were used in PCR reactions with different primers listed in Table 1.

For qRT-PCR, a StepOne Real-Time PCR System (Life Technologies) was used with the primers listed in Table 1. Briefly, each 15-μL reaction mixture contained 25 ng cDNA, 7.5 μL Power SYBR green PCR master mix (Life Technologies), and 300 nM each forward and reverse primer. The reactions were performed with 1 cycle at 95°C for 10 minutes, followed by 40 repeated cycles of 95°C for 15 seconds and 60°C for 1 minute. The 16S rRNA transcript was used as an endogenous control for the qRT-PCR. StepOne Software v2.1 (Life Technologies) was used in data analysis.

### Phenotype microarray analysis

The phenomes of the *A*. *baumannii* ATCC 17978 and its *baeR* mutant strains to various chemical compounds were assayed using the Biolog Phenotype MicroArray (PM) system (Biolog, Hayward, CA). Microplates PM15B, PM17A, and PM19 (http://www.biolog.com/pdf/pm_lit/PM11-PM20.pdf) containing total 72 compounds were used. All phenotypic experiments were performed per the manufacturer’s protocol (Biolog). Following inoculation, all PM plates were incubated at 37°C for 24 hours. The bacterial growth was considered positive where the tetrazolium-based dye (colorless) reduced to formazan (violet), which was determined visually and using a Microplate Reader (Bio-Rad, Hercules, CA).

### Confirmation of the phenotype microarray data using a spot assay

To confirm the phenotype microarray analysis, spot assays were performed. *A*. *baumannii* ATCC 17978 and the *baeR* mutant strains were grown overnight in LB broth with or without kanamycin (37°C, 220 rpm, 16 h). The overnight cultures were sub-cultured in 3 mL LB broth (initial cell density, OD_600_ = 0.3), grown to mid-log phase and harvested through centrifugation 6000 rpm for five minutes. The cell pellets were resuspended in 1 mL phosphate buffered saline (PBS). A ten-fold serial dilution of the bacterial suspension was performed using PBS. The bacteria were then spotted (10 μL/per spot) onto the LB agar plates containing different concentrations of tannic acid (Avantor, Center Valley, PA) and incubated at 37°C for 24 hours.

### BaeR protein purification

BaeR protein purification was performed as previously described [27] with certain modifications. Briefly, the His-tagged *baeR* gene (640 bp) was digested with restriction enzymes *Nhe*I and *Xho*I and ligated to the pET-23a(+) plasmid. The pET-23a(+)-His-BaeR was then transformed into the *Escherichia coli* BL21 (DE3) pLysS strain by heat shock method. A single colony of the transformant was inoculated into LB broth containing ampicillin (50 μg/mL) as well as chloramphenicol (30 μg/mL) and incubated at 37°C overnight (16 hours, with shaking 220 rpm). Cells from the overnight culture were sub-cultured (1:100 dilution) to a fresh LB broth, grown to OD_600_ ~0.6, which was followed by adding 0.5 M isopropyl β-D-1-thiogalactopyranoside (IPTG) (final concentration 0.5 mM); the samples were then further incubated for 4 hours at 37°C and 180 rpm. Cell pellets were collected using centrifugation at 3000 x g and 4°C for 20 minutes, re-suspended in lysis buffer (20 mM NaPB [pH 7.5], 500 mM NaCl) containing 1 mg/mL hen egg white lysozyme (Sigma, St. Louis, MO), and incubated at 4°C for 10 minutes. Thereafter, the cells were lysed through sonication. His-tagged BaeR proteins were collected using centrifugation and maintained on ice before use. The His-BaeR proteins seemed to be in inclusion bodies (i.e., protein aggregates). The inclusion bodies were washed with lysis buffer, resuspended in solubilization buffer (50 mM NaPB [pH 8], 1 M NaCl, 8 M urea, 10% glycerol, and 5 mM imidazole), and centrifuged at 3000 x g and 4°C for 10 minutes. The insoluble materials were discarded, and the supernatant was then mixed with Ni-NTA agarose (Qiagen, Valencia, CA) that was equilibrated with the solubilization buffer. After shaking at room temperature for 1 hour and centrifugation (3000 x g, 4°C for 10 minutes), the agarose pellet was washed twice with 40 mL washing buffer (50 mM NaPB [pH 7] and 4 M urea) containing 0.1% triton X-114 and twice with 20 mL washing buffer. The His-BaeR protein was then purified using Nickel chelate agarose. To elute the Ni-chelated BaeR protein, elution buffer (50 mM NaPB [pH 7], 4 M urea, and 250 mM imidazole) was added to the agarose and mixed at room temperature for 20 minutes. All of the eluted supernatants were pooled together and dialyzed with modified elution buffer containing less urea (50 mM NaPB [pH 7] and 3 M urea, followed with the same buffer containing 2M urea) to eliminate the imidazole. The protein concentration was determined using the bicinchoninic acid (BCA) assay. To refold the His-BaeR protein, the solution was mixed with refolding buffer (20 mM NaPB [pH 7.3] and 10 mg/mL n-octyl-b-D-glucopy-ranoside [OG]) at 42°C for 16 hours and concentrated using an Amicon Ultra-4 centrifugal filter units (Millpore, Billerica, MA) in accordance with the manufacturer’s instruction.

### Electrophoretic mobility shift assay (EMSA)

For the EMSA, a lightshift chemiluminescent EMSA kit (Thermo Scientific, Rockford, IL) was used, and the manufacturer’s protocol was followed. We designed appropriate oligonucleotide pairs that included the putative binding regions for each pump gene. DNA labeling was performed using a Biotin 3´ end DNA labeling kit (Thermo Scientific). The DNA probes were prepared through PCR amplification. DNA (20 fmol) and His-BaeR protein (1 μg) were mixed in binding buffer (10 mM Tris-HCl [pH 7.5], 50 mM KCl, and 1 mM DTT) and poly deoxyinosinic-deoxycytidylic acid (poly (dI-dC)) (50 ng/μL). The reaction mixtures (20 μl) were loaded onto a 5% polyacrylamide gel in 0.5 X TBE (45 mM Tris, 45 mM boric acid, and 1 mM EDTA at pH 8.3). The DNA-protein complexes were separated at 100 V for 2 hours in 0.5 X TBE buffer and then transferred to a Biodyne B nylon membrane (Pall Corporation, Port Washington, NY). Crosslinking and detection of the His-labeled DNA-protein complexes were performed using a UV lamp and chemiluminescence, respectively.

### Statistical analysis

Depending on the suitability of the different samples, the difference in susceptibility was analyzed using chi-square or Fisher’s exact test. The differences between two groups of isolates were considered significant at *P* < 0.05. Data entry and analyses were performed using the Statistical Package for the Social Sciences (SPSS) software version 15.0 (SPSS Inc., Chicago, IL, USA).

## Results

### Minimal inhibitory concentration (MIC) determination

The MIC of tigecycline for the wild-type *A*. *baumannii* ATCC 17978 strain, its *baeR* deletion mutant (AB1026), the *baeR*-reconstituted strain (AB1027), and the *baeR*-overexpressed strain (AB1028) were 0.5, 0.25, 0.5 and 1 μg/mL respectively [19]. The MICs obtained with the induced tigecycline-resistant strain ABtc, ABtcm and the clinical tigecycline-resistant strain ABhl1 were 256, 256 and 16 μg/mL [19]. The tigecycline MIC of the clinical strain (ABhl1) after two-week tigecycline induction (ABhl1tc) was 128 μg/mL.

### The influence of the BaeSR TCS on AdeIJK and MacAB-TolC pump gene expression

**Fig 1:**
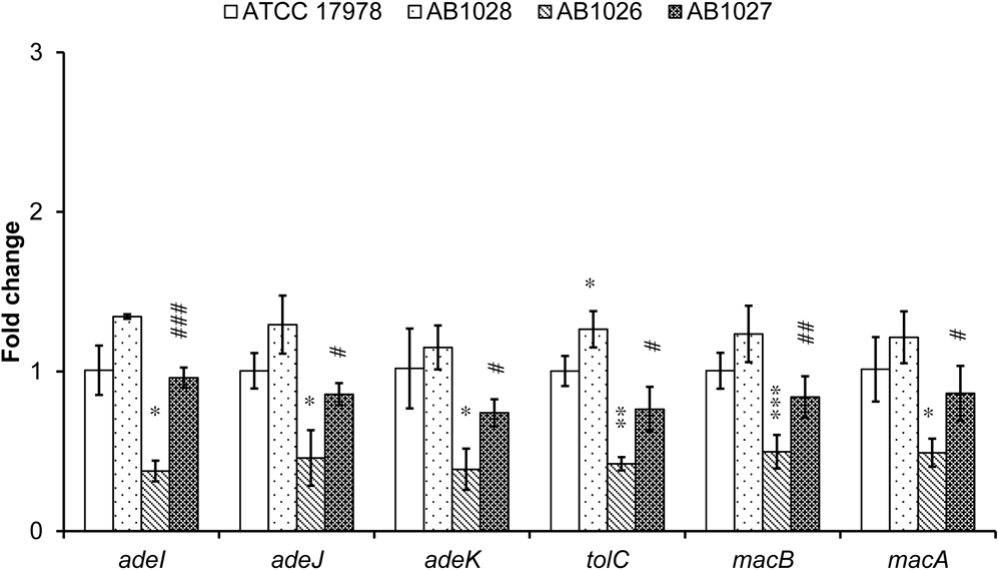
The influence of the BaeSR TCS on AdeIJK and MacAB-TolC pump gene expression. *baeR* deletion in the ATCC 17978 strain significantly reduced *adeI*, *adeJ*, *adeK*, *tolC*, *macA* and *macB* expression. The reduced expression could be restored through trans-complementation with pWH1266-*kan* ^*r*^-*baeR*. Introducing pWH1266-*kan* ^*r*^-*baeR* into the ATCC 17978 strain mildly increased expression of these genes. The results are shown as the means ± SD from three independent experiments. *, *P* < 0.05 and **, *P* < 0.01 and ***, *P* < 0.001 between ATCC 17978 and AB1026; #, *P* < 0.05 and ##, *P* < 0.01 and ###, and *P* < 0.001 between AB1026 and AB1027.

Deleting *baeR* in the ATCC 17978 strain significantly led to 63%, 55%, 63%, 58%, 51% and 52% decrease in gene expression of *adeI*, *adeJ*, *adeK*, *tolC*, *macB* and *macA* respectively (Fig 1). To verify this result, the *baeR* deletion mutant was trans-complemented with pWH1266-*kan* ^*r*^-*baeR*. The gene expression of *adeI*, *adeJ*, *adeK*, *tolC*, *macB* and *macA* in the complemented strains increased 2.6-, 1.9-, 1.9-, 1.8-, 1.7-, and 1.8-fold respectively while being compared with the *baeR* deletion mutant, implicating the reduced expression could be restored through trans-complementation. Introducing pWH1266-*kan* ^*r*^-*baeR* into the ATCC 17978 strain yielded 1.2- to 1.3-fold increases in the expression of each gene without statistical significance except *tolC*.

### The expression of AdeIJK and MacAB-TolC pump genes in the laboratory-induced tigecycline-resistant *A*. *baumannii* and its *baeR* mutant strains

**Fig 2:**
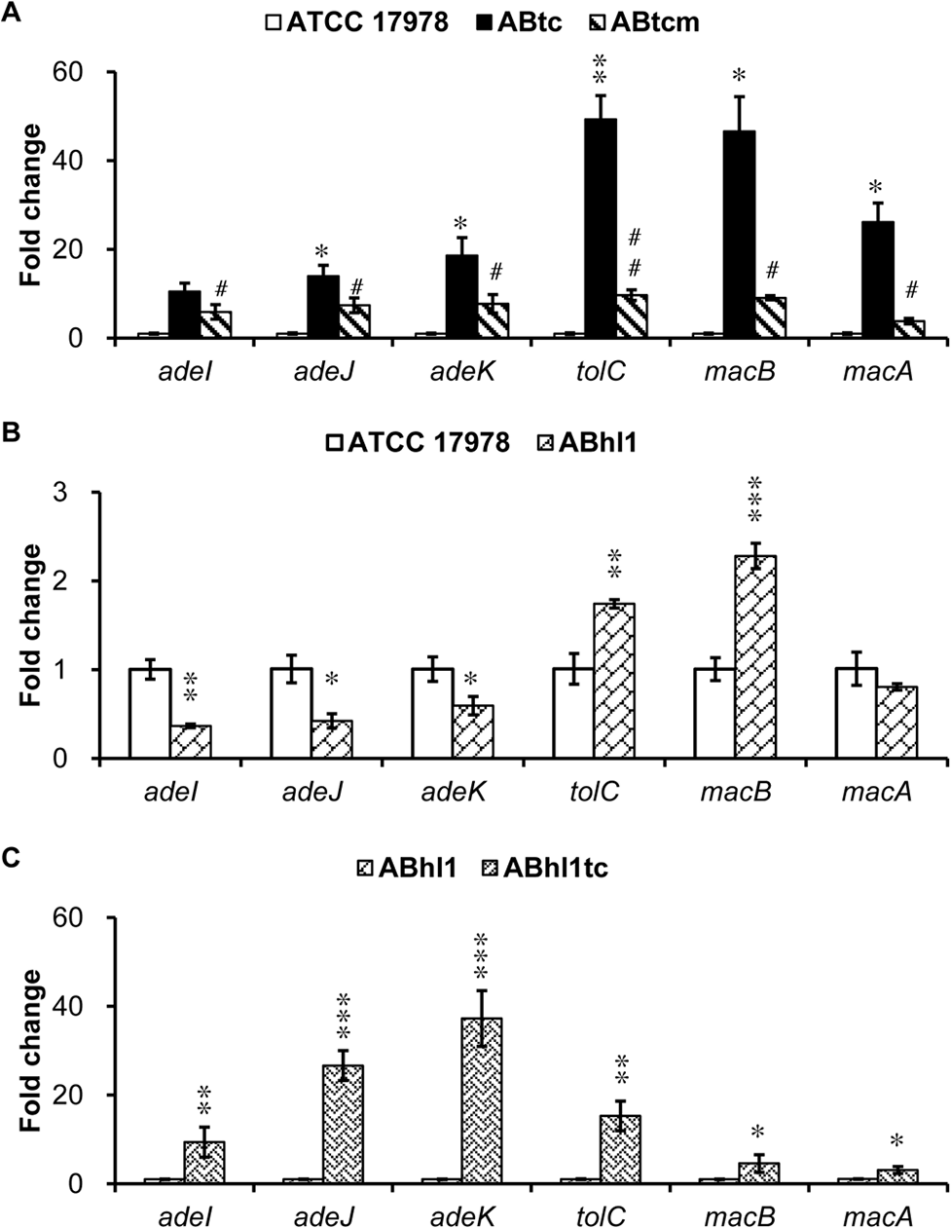
Expression analyses of the AdeIJK and MacAB-TolC pump genes in the laboratory-induced and clinical tigecycline-resistant *A. baumannii* and their *baeR* mutant strains. (A) Gene expression of the AdeIJK pump in ABtc increased compared with the wild-type strain, whereas gene expression of MacAB-TolC in ABtc exhibited a more marked increase after tigecycline induction. The expression levels of *adeI*, *adeJ*, and *adeK* in the ABtcm strain were 44%, 47%, and 59% lower, respectively, than in ABtc, and the expression levels of *tolC*, *macB*, and *macA* in ABtcm were 80%, 81%, and 85% lower, respectively, than in ABtc. (B) The expression levels of *adeI*, *adeJ*, and *adeK* in ABhl1 exhibited a statistically significant decrease compared with the wild-type strain, whereas the expression levels of *tolC* and *macB* increased 1.7 and 2.3-fold, respectively. (C) The gene expression of *adeI*, *adeJ*, *adeK*, *tolC*, *macB* and *macA* in the ABhl1tc strain increased 9.4-, 27-, 37-, 15-, 4.6-, 3.1-fold respectively compared with the ABhl1 strain. The results are displayed as the means ± SD from three independent experiments. *, *P* < 0.05 and, **, *P* < 0.01 and ***, *P* < 0.001 between ATCC 17978 and ABtc or ABhl1. #, *P* < 0.05 and ##, *P* < 0.01 between ABtc and ABtcm.

Gene expression of the AdeIJK pump in tigecycline-resistant *A*. *baumannii* strain (ABtc) increased 11-, 14- and 19-fold compared with the ATCC 17978 strain. However, gene expression of MacAB-TolC in ABtc exhibited an even more marked increase (49-fold for *tolC*, 45-fold for *mac*B and 26-fold for *macA*) after tigecycline induction (Fig 2A). The expression levels of *adeI*, *adeJ*, and *adeK* in the *baeR* mutant strain of ABtc (ABtcm) were 44%, 47%, and 59% decrease respectively compared to that in ABtc. Moreover, the expression levels of *tolC*, *macB*, and *macA* in ABtcm were 80%, 81%, and 85% lower, respectively, than in ABtc. These data confirm that BaeR contributes to AdeIJK and MacAB-TolC regulation, which may be involved in pumping tigecycline in *A*. *baumannii*.

### Expression analyses of AdeIJK and MacAB-TolC pump genes in the tigecycline-resistant *A*. *baumannii* clinical isolate

The expression levels of *adeI*, *adeJ*, and *adeK* in ABhl1 exhibited a statistically significant decrease compared with the wild-type strain (64%, 59%, and 41% reduction) (Fig 2B), whereas the expression levels of *tolC* and *macB* increased 1.7 and 2.3-fold, respectively. To verify these findings, the gene expression of AdeIJK and MacAB-TolC pumps was also examined in the clinical isolate with induced high tigecycline resistance (ABhl1tc). Although the gene expression of *adeI*, *adeJ*, and *adeK* in ABhl1 showed marked decrease compared with the wild-type strain, these genes increased 9.4-, 27-, and 37-folds in the ABhl1tc strain respectively compared with the ABhl1 strain. The increased gene expression of *tolC*, *macB* and *macA* (15-, 4.6-, 3.1-folds respectively) was also observed in ABhl1tc compared with ABhl1.

### Phenotype microarray experiment using the *baeR* mutant

**Fig 3:**
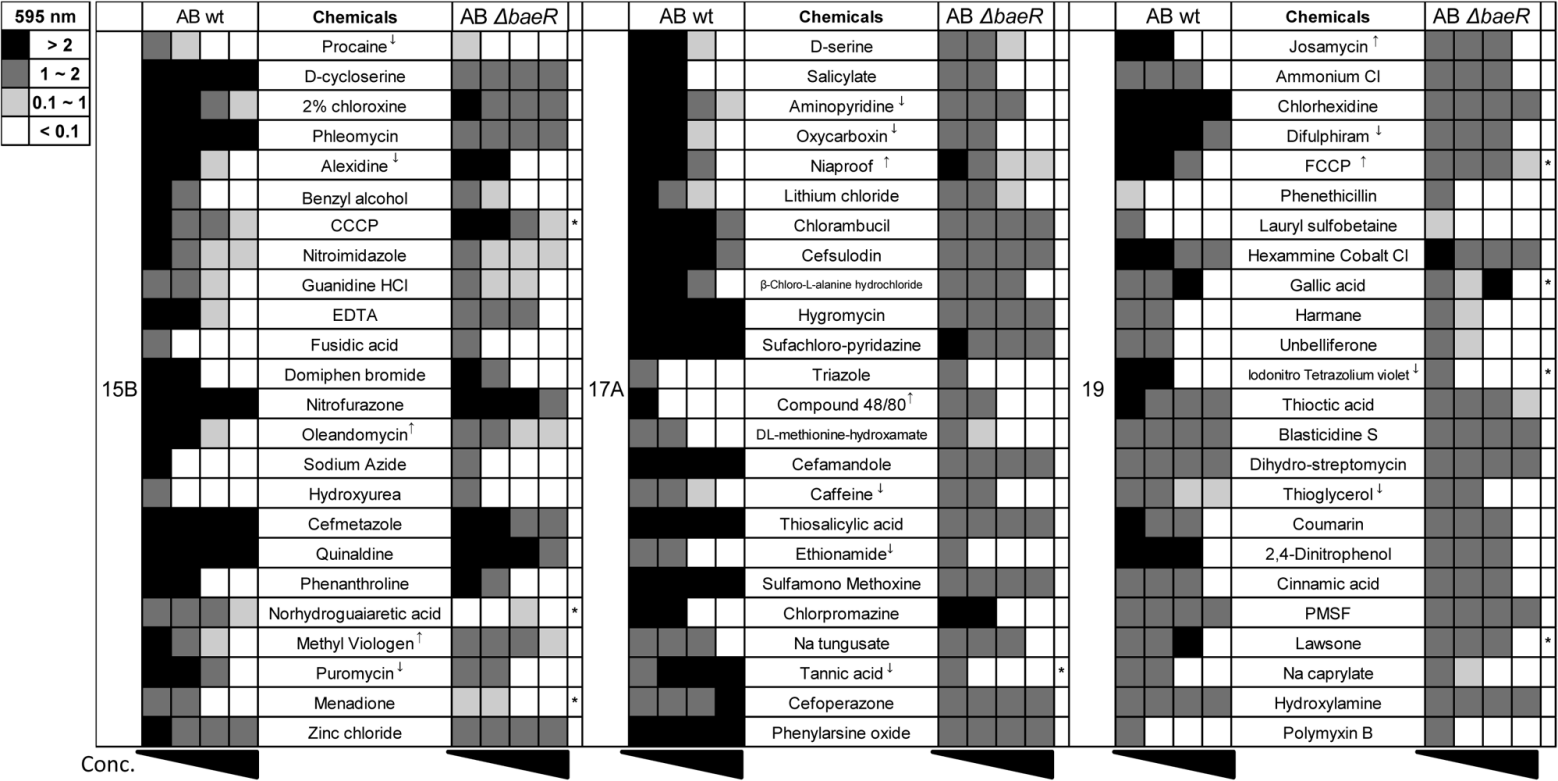
Phenotype microarray using the *baeR* mutant. Differential susceptibility to some of the 72 compounds studied was observed between the ABwt and AB∆*baeR* strains. Four different concentrations of each compound were placed in individual wells next to each other in a row with increasing concentrations from the left to the right. The growth of *A*. *baumannii* was determined by measuring the optical density at 595 nm (OD_595_). Compared to ABwt, growth of AB∆*baeR* was much sensitive with increasing concentrations of several compounds, including procaine, alexidine, and puromycin (in PM 15B); aminopyridine, oxycarboxin, caffeine, ethionamide, tannic acid (in PM17A); and difulphiram, iodonitro tetrazolium violet, and thioglycerol (in PM 19). All these compounds inhibited AB∆*baeR* cell growth were marked with ↓. Among them, tannic acid sensitivity was the most markedly compromised after deleting *baeR*. In contrast, AB∆*baeR* exhibited better tolerance to oleandomycin, methyl viologen, niaproof, compound 48/80, josamycin and FCCP compared to ABwt (All these compounds were marked with ↑). *: the color of the chemical compounds somehow interferes with the observation of bacterial growth.

Susceptibility to some of the 72 studied compounds was examined using the ATCC 17978 and *baeR* mutant strains (referred to as ABwt and AB∆*baeR* in Fig 3). After a 24 h incubation, no AB∆*baeR* cell growth was observed at low concentrations of procaine, alexidine, and puromycin (in PM 15B); aminopyridine, oxycarboxin, caffeine, ethionamide, tannic acid (in PM17A); and difulphiram, iodonitro tetrazolium violet, and thioglycerol (in PM 19) compared with ABwt. In contrast, the ABwt cell growth was inhibited completely at low concentrations of oleandomycin and methyl viologen (in PM15B); niaproof and compound 48/80 in (PM17A); josamycin and FCCP (in PM19) compared with AB∆*baeR*. Although the colors of some chemical compounds, such as tannic acid, cefperazone, and gallic acid, somehow interfere with the determination of bacterial growth by Microplate Reader, with the help of subsequent bacterial cultures, tannic acid resistance was still shown as the most markedly compromised chemical after *baeR* deletion. Therefore, tannic acid was used for further study to determine the relationship between tannic acid and expression of *baeR* as well as pump genes.

### Confirmation of the phenotype microarray results using a spot assay

**Fig 4:**
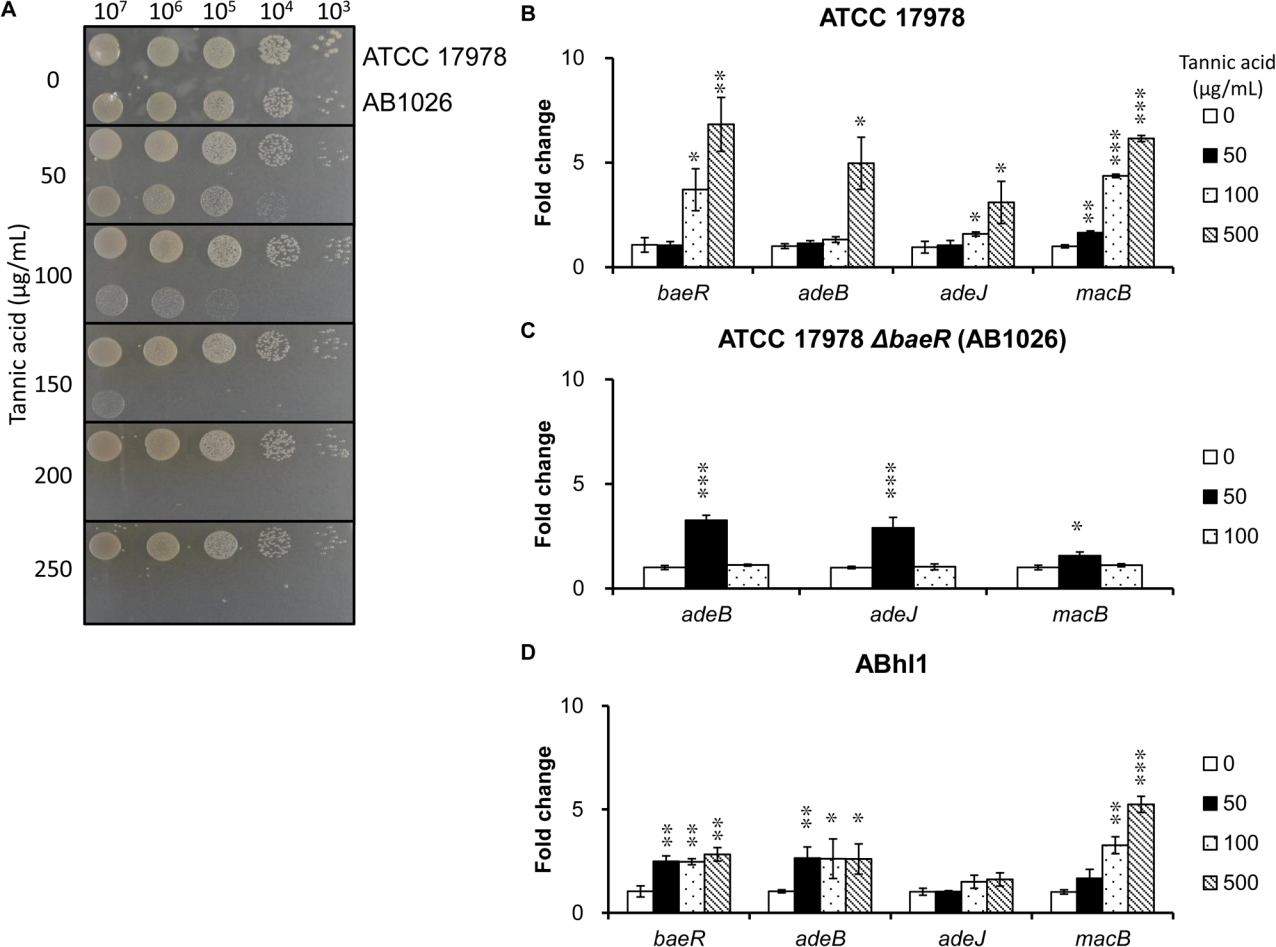
Spot assays and gene expression analyses after tannic acid exposure. (A) Spot assay. The wild-type strain exhibited better tolerance to tannic acid than the *baeR* mutant strain. (B) The gene expression levels of the *baeR* and pump genes in the wild-type strain. At 50 μg/mL tannic acid, only *macB* showed a statistically significant increase in the wild-type strain. Through increasing medium tannic acid up to 100 μg/mL, *baeR*, *adeJ* and *macB* exhibited increased gene expression. If the medium contained tannic acid 500 μg/mL, the expression of each gene investigated in the wild-type strain increased dramatically. (C) The gene expression levels of the pump genes in the *baeR* mutant strain. The gene expression of *adeB*, *adeJ*, and *macB* in the *baeR* mutant strain increased significantly at the tannic acid concentration 50 μg/mL. Through increasing the tannic acid concentration to 100 μg/mL, expression of each gene decreased to levels without tannic acid exposure. (D) The gene expression levels of the *baeR* and pump genes in the clinical strain ABhl1. The gene expression of *baeR*, *adeB*, and *macB* demonstrated a statistically significant increase upon being exposed to 100 and 500 μg/mL tannic acid. The results are shown as the means ± SD from three independent experiments. *, *P* < 0.05 and, **, *P* < 0.01 and ***, *P* < 0.001.

The ATCC 17978 strain can tolerate tannic acid as high as 250 μg/mL (Fig 4A). However, we did not observe growth of 20 μL 10^3^ cells/mL *baeR* mutant bacterial solution in the LB plate containing 50 μg/mL tannic acid, whereas 100 μg/mL tannic acid fully inhibited 10^4^ cells/mL *baeR* mutant strain. In the presence of 150 μg/mL tannic acid, no diluted bacterial solutions exhibited growth, except 10^7^ cells/mL *baeR* mutant strain, which exhibited slight growth With an increasing tannic acid concentration, none of the studied bacterial *baeR* mutant strain solutions grew.

### Gene expression analyses of *baeR* and pump genes after tannic acid exposure

To understand the AdeAB, AdeIJK, MacAB-TolC, and BaeRS system responses upon tannic acid exposure, qRT-PCR of *adeB*, *adeJ*, *macB* and *baeR* was performed for the ATCC 17978, its *baeR* mutant strain and the clinical strain ABhl1. At 50 μg/mL tannic acid, only *macB* exhibited a statistically significant increase (1.7-fold) in the *A*. *baumannii* ATCC 17978 strain (Fig 4B). With an increase in tannic acid up to 100 μg/mL, *baeR*, *adeJ* and *macB* exhibited increased gene expression (3.7-, 1.6- and 4.4-fold respectively) and were compared with samples that were not exposed to tannic acid. If the medium contained 500 μg/mL tannic acid, the expression of the genes investigated in the ATCC 17978 strain, *baeR*, *adeB*, *adeJ*, and *macB* clearly increased (6.8-, 5.0-, 3.1- and 6.2-fold respectively).

However, the *baeR* mutant strain cannot survive in the medium with 500 μg/mL tannic acid. The gene expression for *adeB*, *adeJ*, and *macB* in the *baeR* mutant strain significantly increased (3.3-, 2.9- and 1.6-fold respectively) at 50 μg/mL tannic acid (Fig 4C). Upon increasing the tannic acid concentration to 100 μg/mL, the gene expression decreased to the levels without tannic acid exposure.

In the clinical strain ABhl1, the *baeR* gene expression increased 2.5 to 2.8 folds and the *adeB* gene expression increased 2.6-fold upon being exposed to 50, 100 and 500 μg/mL tannic acid (Fig 4D). The gene expression of *macB* increased 3.3-fold at 100 μg/mL tannic acid and increased 5.2-fold at 500 μg/mL tannic acid. However, the *adeJ* gene expression did not show significant fold change while being exposed to tannic acid.

### Electrophoretic mobility shift assays

EMSA was performed using different combinations of 10 fmol/μL biotin-labeled DNA (243-bp, 144-bp, and 329-bp DNA fragments upstream of *adeA*, *adeI* and *macA*, respectively), 2 pmol/μL unlabeled DNA and 0.05 μg/μL purified His-BaeR. Used as a nonspecific competitor, 50 ng/μL Poly (dI-dC) was also added. Adding His-BaeR protein to each biotin-labeled DNA probe did not cause band shift compared to the reaction with the probe only (no His-BaeR added) (S1 Fig). Therefore, we concluded that no protein-DNA complexes formed between His-BaeR protein and each of the *adeA*, *adeI* and *macA* promoter region. In addition to the purified His-BaeR, we also used total protein extract from *A*. *baumannii* ATCC 17978 and it’s *baeR*-deletion mutant. Band shift was observed from the reaction containing protein extract and each of the *adeA*, *adeI* and *macA* promoter region. The band shift did not appear in the reaction lacking of protein extract. Moreover, the band shift occurred in the reaction using protein extract from either ATCC 17978 or the *baeR*-deletion strains. These results suggest that *adeA*, *adeI* and *macA* genes are controlled by regulator other than BaeR.

## Discussion

Bacterial two-component regulatory systems (TCSs), which consist of a sensor histidine kinase and a response regulator, facilitate changes in gene expression in response to environmental stimuli [34]. To ensure cell survival in harsh conditions, such as being exposed to hazardous chemicals, histidine kinase can sense the environmental signal and autophosphorylate. The phosphate is then transferred to an aspartic acid residue of the corresponding response regulator. The phosphorylated response regulator can thus elicit many diverse responses, including enhancing its DNA binding ability to modulate target gene expression [35]. Nineteen TCSs were identified in a clinical isolate of multidrug resistant *A*. *baumannii* by Adams et al., most of which were also in other clinical isolates, including *A*. *baumannii* AYE as well as ACICU, and 17 TCSs are conserved in the *A*. *baumannii* ATCC 17978 strain. However, functional studies on the TCSs and their downstream target genes are currently limited in *A*. *baumannii*. Only the TCSs PmrAB [36,37], BfmRS [38,39], AdeRS [15,16], BaeSR [19] and GacSA [40] have been characterized. Colistin resistance in *A*. *baumannii* can be due to adding phosphoethanolamine to cell wall lipopolysaccharide, which is mediated by *pmrAB* mutations [36]. Over-expression of the AdeABC efflux pump stimulated by the mutated AdeRS results in antimicrobial resistance, including tigecycline resistance, in multidrug-resistant *A*. *baumannii* (MDRAB) [16]. BfmRS TCS is related to biofilm formation [38] and mediates virulence in *A*. *baumannii* [39], whereas GacSA acts as a global virulence regulator and involves pili formation, motility and biofilm structure [40]. Nevertheless, the role of *A*. *baumannii* TCSs in disposing environmental chemical compounds has not been clarified.

Similar to other TCSs, BaeSR can detect environmental signals and respond by altering the bacterial envelope [25]. We have shown that the BaeSR regulons not only respond to high osmotic stress but also influence the tigecycline susceptibility of *A*. *baumannii* through positively regulating the RND efflux pump genes *adeA* and *adeB* [19]. In this study, an additional two transport systems, AdeIJK and MacAB-TolC, may have also been regulated by BaeSR because *baeR* deletion reduced gene expression in the two pump systems. This result is consistent with transcriptional data from an LPS-deficient *A*. *baumannii* strain, which showed the increased expression of BaeSR, AdeIJK and MacAB-TolC [30].

Tigecycline is a glycycline and is one of the few available effective antibiotics for MDRAB infections [41]. Several previous studies have demonstrated that tigecycline resistance is mainly facilitated through efflux pumps, including AdeABC [7], AdeIJK [10] and AdeFGH [9]. The AdeABC and AdeIJK efflux systems’ tigecycline resistance is greater than an additive contribution [10]. In this study, qRT-PCR data also demonstrated a potential role for AdeIJK and MacAB-TolC in tigecycline resistance of the laboratory-induced tigecycline-resistant *A*. *baumannii* strains (ABtc and ABhl1tc). In 2011, Coyne et al. declared that AdeABC was the only system involved in clinical isolate tigecycline resistance [42]. Besides, AdeIJK is considered to play a role in the intrinsic low-level resistant phenotype of *A*. *baumannii* [10] and over-expression of this pump in *A*. *baumannii* is toxic, suggesting the presence of a tight regulation mechanism to maintain low expression levels of AdeIJK [43] in ABhl1 compared with the wild type strain.

In addition to conferring clinically relevant resistance to antibiotics, these multidrug-resistance efflux pumps encoded by bacteria can also confer resistance to natural substances produced by the host [13]. To elucidate the TCS BaeSR response after being exposed to environmental chemical compounds through the efflux pumps, we used a phenotypic microarray. *A*. *baumannii* was vulnerable to certain chemicals, especially tannic acid, after *baeR* deletion. Tannins comprise a large group of natural products distributed in the vegetable kingdom and has been classified into two groups, condensed and hydrolysable [44]. Tannic acid has long been used as a topical agent for burn wounds. Tannic acid was further proposed as an adjuvant therapy with β-lactam antibiotics for *Staphylococcus aureus* infections [45]. Susceptibility of MDRAB to a variety of antibiotics was also enhanced in the presence of tannic acids [46]. The antioxidant capacity and antimicrobial activity of tannic acid can be enhanced by thermal processing [47]. Despite tannin antimicrobial activities, many tannin-resistant bacteria have been isolated. However, the mechanisms underlying this resistance remain unclear. The TCS BaeSR has been thought to mediate tannic acid resistance through up-regulating the multidrug transporter-encoding operon *mdtABCD* in *E*. *coli* [26]. In our previous and present studies, we found that both tigecycline and tannic acid resistance are associated with the BaeR regulon in *A*. *baumannii* using qRT-PCR data. *baeR* gene deletion influenced gene expression for the AdeAB, AdeIJK and MacAB-TolC pump systems. Increased gene expression of *adeB*, *adeJ*, and *macB* after being exposed to tigecycline or high concentration of tannic acid was also demonstrated. These results suggested a modified role for BaeR, as Appia-Ayme et al. proposed, in up-regulating certain pump systems in response to specific envelop damaging agents as a belt and braces approach to protect the cell through waste disposal [23].

As shown in Fig 4B, the *baeR* gene expression did not increase upon exposure to 50 μg/mL tannic acid, whereas *macB* expression showed a slight increase. In addition, similar to the *baeR* gene, the *adeB* and *adeJ* gene expression was also no change in the presence of 50 μg/mL tannic acid. These results suggest that *macB* gene expression may be controlled by a regulator other than BaeR. Interestingly, the expression of *adeB*, *adeJ* and *macB* pump genes was increased in the *baeR*-deletion background with 50 μg/mL tannic acid, whereas decreased with 100 μg/mL tannic acid (Fig 4C). One possible explanation is that the *A*. *baumannii* efflux pumps were mainly controlled by local regulators (e.g., AdeRS controls *adeAB* and AdeN controls *adeIJK*) upon exposure to low tannic acid concentrations. However, the BaeR regulon includes these pump genes at higher tannic acid concentrations. This hypothesis highlights the complex transcription regulatory networks in *A*. *baumannii* and requires further study. Moreover, EMSAs of the interaction between BaeR-His6 and the *adeA*, *adeI* and *macA* promoter regions did not exhibit direct binding, which implies that their mutual interaction is possibly indirect. One previous paper indicates that BaeSR is part of a cross-regulation system that includes the PhoBR and CreBC TCSs in *E*. *coli* [29]; it suggests that constructing the BaeSR mutants may not only affect the expression levels of genes under direct control of BaeSR but may disrupt a complex regulatory network.

Despite the few results for TCS in *A*. *baumannii*, it remains a possible target for therapeutics, which is supported by the conclusion from Gram-positive pathogenic bacteria [48]. A class of antibacterial agents that inhibit two-component signal transduction systems was developed in the laboratory and may represent a breakthrough in antibacterial therapy [49]. Moreover, a small molecule adjuvant was found capable of suppressing colistin resistance in MDRAB by interfering with the expression of TCS PmrCAB [50]. In conclusion, *A*. *baumannii* can use the TCS BaeSR in disposing chemicals, such as tannic acid and tigecycline, through regulating efflux pumps without direct DNA binding. Our findings may help to understand TCS and efflux pumps-associated antimicrobial resistance mechanisms and provide a basis for the future development of antimicrobials against drug-resistant *A*. *baumannii*.

## Supporting Information

S1 Fig Electrophoretic mobility shift assays. (PDF)
