# Supplementary material for: The Role of the Two-Component System BaeSR in Disposing Chemicals through Regulating Transporter Systems in Acinetobacter baumannii
Source: PLoS One. 2015 Jul 10;10(7):e0132843. doi: 10.1371/journal.pone.0132843 (PMC4498774; doi:10.1371/journal.pone.0132843)
Supplement: S1 Fig — (PDF) [file pone.0132843.s001.pdf]

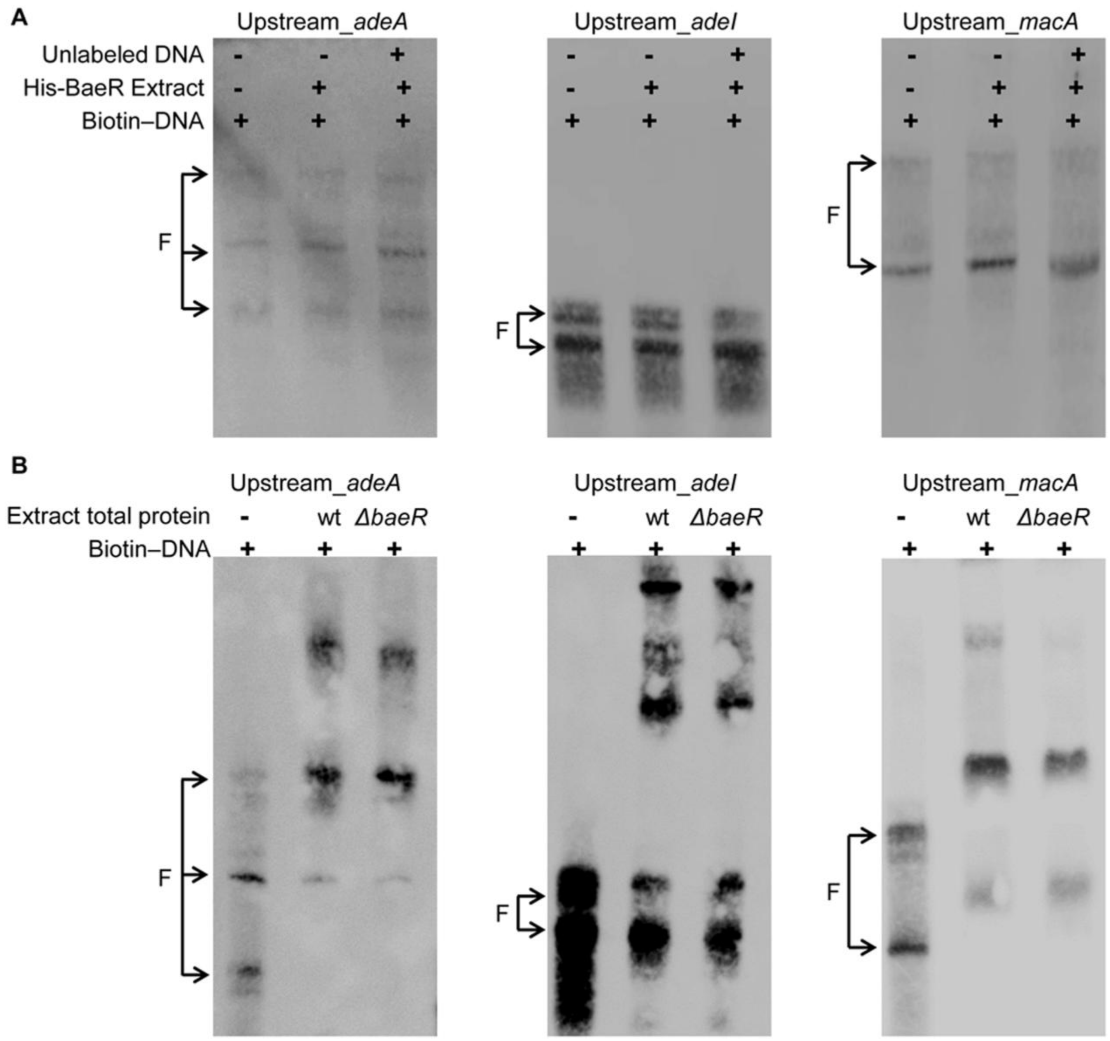

Electrophoretic mobility shift assays. EMSAs were performed using purified His-BaeR and the 243-bp, 144-bp, and 329-bp DNA fragments upstream of *adeA*, *adeI* and *macA*, respectively. (A) Biotin-labeled DNA added with His-BaeR did not lead to band shift by comparison with no His-BaeR added. (B) In the presence of total protein from the *A. baumannii* ATCC 17978 and its *baeR* mutant strains, the DNA probe associated with the protein in a complex that was observed based on the upward shift of the migrating bands. F, free DNA probe.
